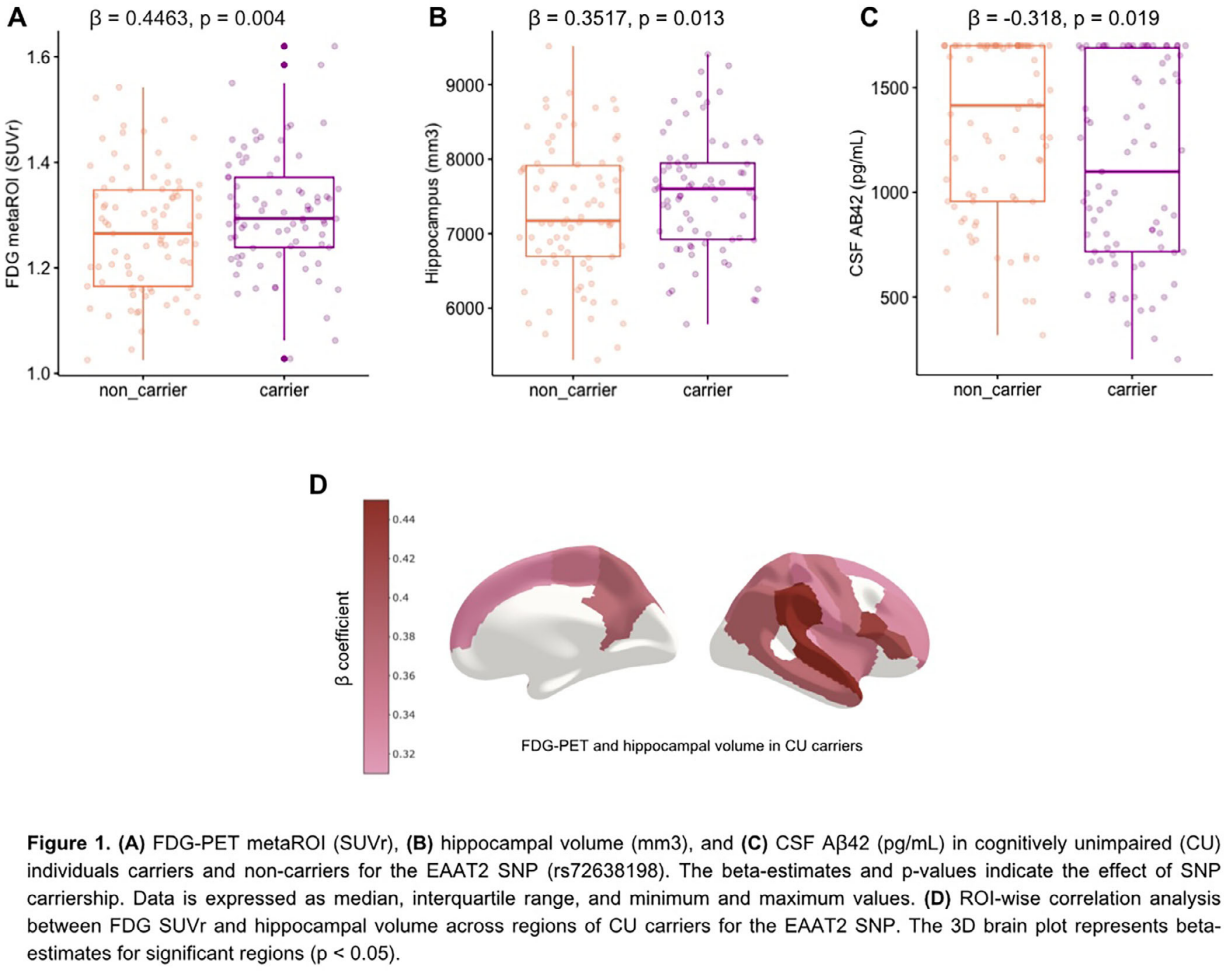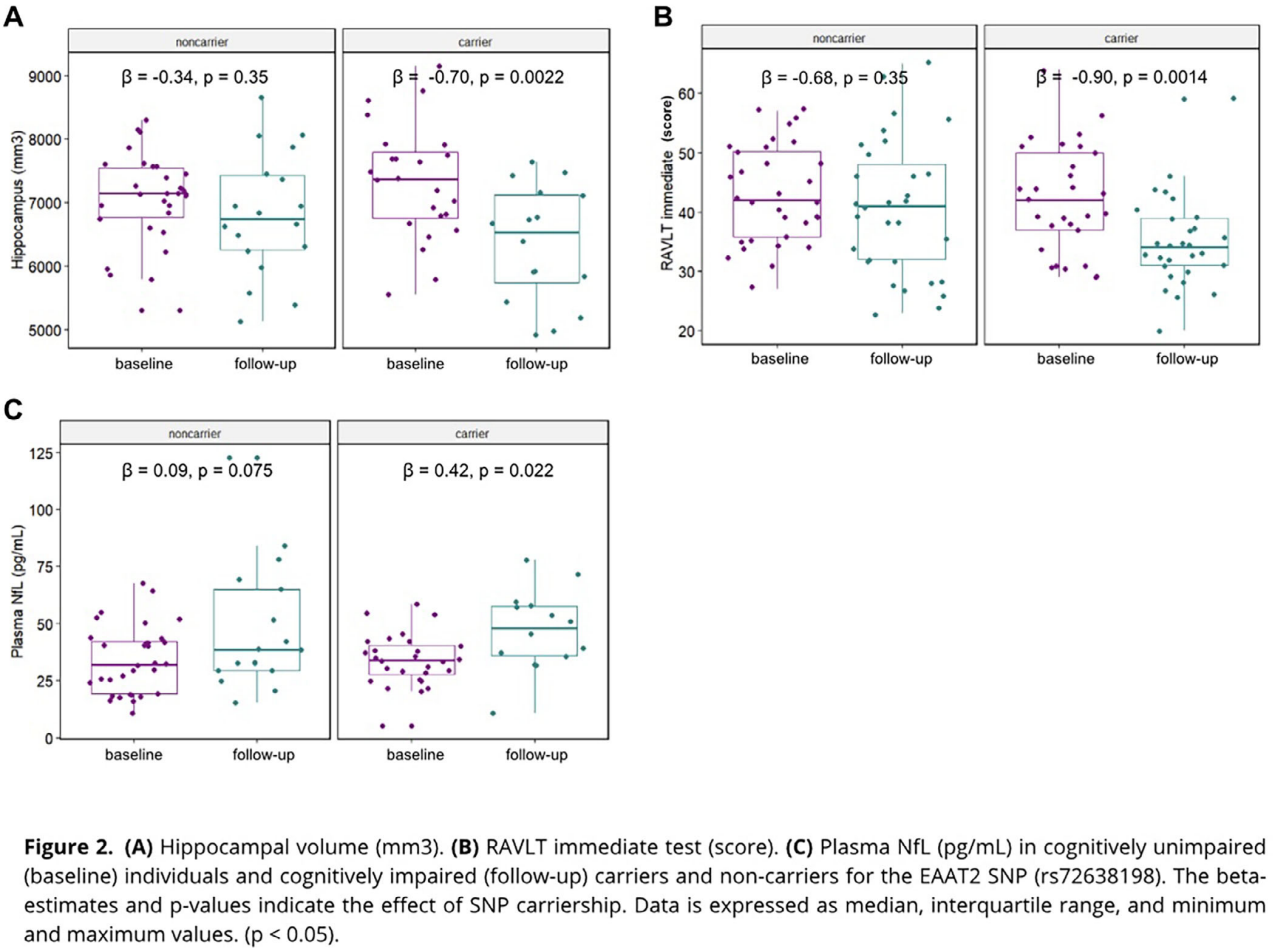# Impact of a single nucleotide polymorphism in the excitatory amino acid transporter 2 (EAAT2) on Alzheimer's disease progression

**DOI:** 10.1002/alz70856_105612

**Published:** 2026-01-08

**Authors:** Mariana Radaelli Schmaedek, Christian Limberger, Gabriel Colissi Martins, Gabriel Lermen Hoffmeister, Ramon Bertoldi de Souza, Roberta dos Santos de Oliveira, Marco Antônio De Bastiani, Eduardo R. Zimmer

**Affiliations:** ^1^ Universidade Federal do Rio Grande do Sul, Porto Alegre, Rio Grande do Sul, Brazil; ^2^ University of Cologne, Cologne, Germany; ^3^ Brain Institute of Rio Grande do Sul (InsCer), PUCRS, Porto Alegre, Rio Grande do Sul, Brazil; ^4^ McGill Centre for Studies in Aging, Montreal, QC, Canada

## Abstract

**Background:**

The complex interplay between brain metabolism and Alzheimer's disease (AD) highlights the vulnerability of neuronal energy pathways and the importance of neuron‐to‐astrocyte metabolic cooperation. Astrocytic glutamate uptake via the excitatory amino acid transporter 2 (EAAT2) stimulates glycolysis, facilitating the conversion of glucose to lactate, and serving as an energy substrate for neurons. This process is called the “astrocyte‐neuron lactate shuttle” (ANLS). The potential contribution of ANLS dysfunction in AD progression remains uncertain. Here, we investigate how carrying a single nucleotide polymorphism (SNP) of the glutamate transporter EAAT2 impacts individuals before and after AD clinical conversion.

**Method:**

We evaluated 493 cognitively unimpaired (CU) and impaired (CI) individuals from ADNI, with available FDG‐PET, MRI, CSF biomarkers, and EAAT2 genotyping (rs72638198). This variant was selected as the most significant SNP influencing FDG‐PET in a previous analysis. We performed linear mixed‐models to analyze the influence of SNP carriership on AD biomarkers, adjusting for age, sex, APOE4 status, Aβ‐ and tau‐status (*p* < 0,05). Further analysis was performed with 62 CU individuals analyzed at the moment of clinical conversion.

**Result:**

CU carriers of the EAAT2 SNP presented brain hypermetabolism and increased hippocampal volume (Figure 1A‐B), which was positively associated with FDG‐PET across cortical regions, as observed in an ROI‐wise correlation analysis (Figure 1D). These individuals also showed lower levels of CSF Aβ42 (Figure 1C). Further investigation was conducted following their conversion to CI, revealing that SNP carriers had a greater decrease in hippocampal volume, higher levels of plasma NfL, and a lower score in the RAVLT immediate test (Figure 2).

**Conclusion:**

CU carriers exhibited changes that may indicate an early alteration profile, as brain hypermetabolism and greater hippocampal volume might be compensatory mechanisms for brain injury in preclinical stages. The lower CSF Aβ42 levels align with Aβ plaque accumulation, possibly indicating early amyloid pathology. At conversion to CI, carriers exhibited more pronounced changes than non‐carriers, indicating that EAAT2 SNP carriership may significantly impact AD progression.